# Does aerobic exercise effect pain sensitisation in individuals with musculoskeletal pain? A systematic review

**DOI:** 10.1186/s12891-022-05047-9

**Published:** 2022-02-03

**Authors:** Lynn Tan, Flavia M Cicuttini, Jessica Fairley, Lorena Romero, Mahnuma Estee, Sultana Monira Hussain, Donna M Urquhart

**Affiliations:** 1 Department of Epidemiology and Preventive Medicine, School of Public Health and Preventive Medicine, Monash University, 553 St Kilda Rd, Melbourne, Victoria 3004 Australia; 2 The Ian Potter Library, The Alfred Hospital, Melbourne, Victoria 3004 Australia

**Keywords:** Aerobic exercise, Pain sensitisation, Musculoskeletal pain, Systematic review, Pressure pain threshold

## Abstract

**Background:**

Pain sensitisation plays a major role in musculoskeletal pain. However, effective treatments are limited, and although there is growing evidence that exercise may improve pain sensitisation, the amount and type of exercise remains unclear. This systematic review examines the evidence for an effect of aerobic exercise on pain sensitisation in musculoskeletal conditions.

**Methods:**

Systematic searches of six electronic databases were conducted. Studies were included if they examined the relationship between aerobic physical activity and pain sensitisation in individuals with chronic musculoskeletal pain, but excluding specific patient subgroups such as fibromyalgia. Risk of bias was assessed using Cochrane methods and a qualitative analysis was conducted.

**Results:**

Eleven studies (seven repeated measures studies and four clinical trials) of 590 participants were included. Eight studies had low to moderate risk of bias. All 11 studies found that aerobic exercise increased pressure pain thresholds or decreased pain ratings in those with musculoskeletal pain [median (minimum, maximum) improvement in pain sensitisation: 10.6% (2.2%, 24.1%)]. In these studies, the aerobic exercise involved walking or cycling, performed at a submaximal intensity but with incremental increases, for a 4-60 min duration. Improvement in pain sensitisation occurred after one session in the observational studies and after 2-12 weeks in the clinical trials.

**Conclusions:**

These findings provide evidence that aerobic exercise reduces pain sensitisation in individuals with musculoskeletal pain. Further work is needed to determine whether this translates to improved patient outcomes, including reduced disability and greater quality of life.

**Supplementary Information:**

The online version contains supplementary material available at 10.1186/s12891-022-05047-9.

## Background

Musculoskeletal pain is a leading global health problem. One in three people worldwide live with a musculoskeletal condition, which is characterised by pain and disability, leads to reduced quality of life, and results in a huge economic burden [1–3]. While chronic musculoskeletal pain includes a heterogeneous group of conditions, which are characterised by pain in different regions/joints and with varying pathogeneses [4], pain sensitisation has been identified as a significant component of these conditions [5]. Pain sensitisation, according to the International Association for the Study of Pain, is defined as “an increased responsiveness of nociceptive neurons to their normal input, and/or recruitment of a response to normally subthreshold inputs” [6]. Pain sensitisation is common in musculoskeletal pain cohorts, with reports of 30% of individuals with osteoarthritis (OA) [7], and 66% of low back pain patients exhibiting high pain sensitivity [8]. Moreover, there is growing evidence highlighting the association between pain sensitisation and significant disability and poor quality of life in musculoskeletal conditions [9, 10]. Currently therapies targeting pain sensitisation are limited [11, 12], with unclear evidence for the efficacy of commonly used interventions, such as off label (unapproved indications) tricyclic antidepressants, as well as the major issue of significant side effects associated with opioid use, including addiction, fatality and hyperalgesia [13–17].

 Exercise is recommended for the management of musculoskeletal pain in national and international treatment guidelines [18–21]. While there are many types of exercise, including aerobic, strengthening, and stretching, there are limited data available to indicate which type of exercise and dosage is effective for reducing pain sensitisation. Aerobic exercise, which typically includes walking, stationary cycling, or stepping, involves physical exercise of low to high intensity that depends primarily on the aerobic energy-generating process, i.e. the use of oxygen to meet energy demands [22, 23]. It has been proposed that aerobic exercise may reduce pain sensitisation by activating descending pain inhibitory mechanisms and/or endogenous opioid and cannabinoid systems in individuals with musculoskeletal pain [24–26].

Narrative reviews and a meta-analysis have been conducted to determine whether aerobic exercise effects pain sensitivity in both healthy individuals and those with chronic pain [22, 27–30]. A meta-analysis by Naugle et al. (2012) found that in healthy individuals a bout of aerobic exercise resulted in a reduction in sensitivity to painful stimuli, termed as ‘exercise-induced hypoalgesia’. However, the review also included studies examining chronic pain and found conflicting findings, with aerobic exercise producing both a hyper and hypoalgesic response [27]. While these contrasting responses may be explained by half of the included studies examining the response of individuals with fibromyalgia [31, 32] and chronic fatigue syndrome [33] and finding a hyperalgesic effect, the role of aerobic exercise in chronic musculoskeletal conditions, such as low back and knee pain, is still unclear. Thus, the research questions to be examined in this systematic review were:

1. Does aerobic exercise effect pain sensitisation in individuals with musculoskeletal pain? If so, what effect does it have?
2. What type and dosage of aerobic exercise is associated with a change in pain sensitisation?

## Methods

 This systematic review was conducted according to the 2020 PRISMA guidelines [34].

### Search strategy

We performed electronic searches of six databases, including OVID MEDLINE, OVID Embase, OVID EBM Reviews Cochrane Central Register of Controlled Trials, OVID PsycINFO, CINAHL and SPORTDiscus, from database inception to 26th March 2021. An initial search for studies was conducted in OVID Medline, and an analysis of text words and subject terms was then used to develop the search strategy, with subject classification systems investigated for each database. The final searches of all six databases, covering the key concepts of aerobic physical activity, pain sensitisation and musculoskeletal pain, were performed using the appropriate specifications for each database. The comprehensive search strategy for OVID Medline is shown in the Supplementary File 1. The searches were limited to studies of adults (≥18 years of age) and those published in the English language. We also searched the reference lists of systematic and narrative reviews and meta-analyses in the field, as well as the studies included in this review.

### Study identification

Titles and abstracts were assessed by two authors (LT, DU) for relevance using Covidence and the full texts were retrieved for relevant studies. We included studies that examined the role of aerobic exercise on pain sensitisation in musculoskeletal pain cohorts (Table 1). Aerobic exercise was defined as physical activity, such as walking, cycling and stepping, which involves large muscle groups and the consumption of oxygen to generate energy [22, 23]. Pain sensitisation was measured using validated, quantitative measures, such as pressure and thermal pain thresholds (PPT/TPT), where the minimum mechanical force or heat applied that induces pain is determined, and pressure and thermal pain ratings, where perceived pain is recorded on a pain rating instrument (e.g. visual analogue scale) during the pressure or thermal stimulus respectively [35–37]. Musculoskeletal pain was defined as pain caused by varying pathogeneses in muscles, bones, joints and associated tissues such as tendons and ligaments [38]. We excluded studies that examined the following exercise types; eccentric, strengthening, resistance, weight training, stabilization, postural correction, stretching and/or mobility, and neuromobilisation. Studies that examined Yoga, Tai Chi, Jyoti, Qigong, Pilates, and Zumba and did not specify an aerobic nature were also excluded, as were studies that assessed the effect of aerobic exercise on pain intensity, rather than pain sensitivity.

**Table 1:** Inclusion criteria

*Design*
- Observational studies
- Randomised or quasi-randomised trials
- English language studies
*Participants*
- Individuals with musculoskeletal pain, but not specific subgroups, such as fibromyalgia, chronic fatigue syndrome, and whiplash, that have been shown to differ in their response to pain.
*Intervention*
- Aerobic physical activity
*Comparisons*
- Aerobic physical activity versus control
- Aerobic physical activity and intervention A versus intervention A
*Outcome measures*
- Pain sensitisation, including pressure or thermal pain thresholds or pain ratings

While we included clinical trials that examined aerobic exercise combined with another intervention compared to that intervention alone, we excluded trials that examined the effectiveness of a combination of treatments or compared aerobic exercise with another intervention. Specifically, we excluded studies that examined the following conditions; complex regional pain syndrome, fibromyalgia, rheumatoid arthritis, chronic fatigue syndrome, headache, migraine, temporomandibular joint disorders, myofascial pain, whiplash and inflammatory arthritis.

### Data extraction

Two authors (LT and DU) extracted and tabulated information on the characteristics of the included studies, including; demographics of the cohort, type of musculoskeletal pain, study inclusion and exclusion criteria, measurement of pain sensitisation, types and dosage of the aerobic exercise, and the results and conclusions of the studies. PPT was defined as the minimum force applied which induces pain and thermal pain threshold was defined as the minimal heat which produces pain, signifying a quantitative value related to the mechanical sensitivity to pain of deep structures [35, 36]. These validated measures have widespread use in evaluating muscular and joint tenderness associated with musculoskeletal conditions, and for the diagnosis and efficacy analysis of management strategies [39, 40]. An increase in pressure or thermal pain thresholds or decrease in pressure or thermal pain ratings is considered to signify hypoalgesia and a reduction in pain sensitisation [37, 41].

### Risk of bias

Risk of bias was assessed using methods adapted from the Cochrane Collaboration for observational studies and randomised controlled trials [42, 43]. The assessment was performed by two reviewers (JF, LT) independently and where consensus could not be achieved, a judgement was made by DU and SMH. We assessed the internal and external validity of the observational studies based on seven items. Each item was assessed as “yes”, “no”, “unclear” or “not applicable” and these contributed to an overall assessment of the risk of bias for each study. The overall assessment for the study was ‘low’ if the answer was “yes” to all items, ‘moderate’ if the answer was “no” or “unclear” to one or two items, or ‘high’ if the answer was “no” or “unclear” to more than two items. To assess the risk of bias of the randomised controlled trials, eight criteria were used to assess the internal validity of the trials. The criterion were scored as “yes”, “no”, or “unclear”. Low risk of bias was defined as fulfilling six or more of the eight quality criteria (i.e. obtaining a ’yes’ score to at least six criteria).

### Clinical heterogeneity

We assessed the clinical heterogeneity of the trials in this review using the Clinical Diversity In Meta-analyses (CDIM), a new tool for assessing clinical diversity between trials in systematic reviews and meta-analyses of interventions [44]. Two authors (LT, DMU) assessed clinical diversity across the four domains of setting, population, intervention and outcome, and a total consensus score from 0 to 22 was calculated, with 0 indicating no clinical heterogeneity and 22 high levels of heterogeneity. While there is no validated tool for assessing clinical heterogeneity between observational studies, we used and adapted the same 4 domains from the CDIM to examine whether the clinical data from observational studies were homogeneous.

### Best evidence synthesis

A best evidence synthesis was used to summarise the data, as it was not possible to perform a meta-analysis due to the clinical heterogeneity between the studies. The studies were ranked according to their design, with clinical trials considered to be a higher level of evidence than observational studies. Both the study design and risk of bias scores were considered when determining the evidence available.

## Results

### Flow of the studies through the review

**Fig. 1:**
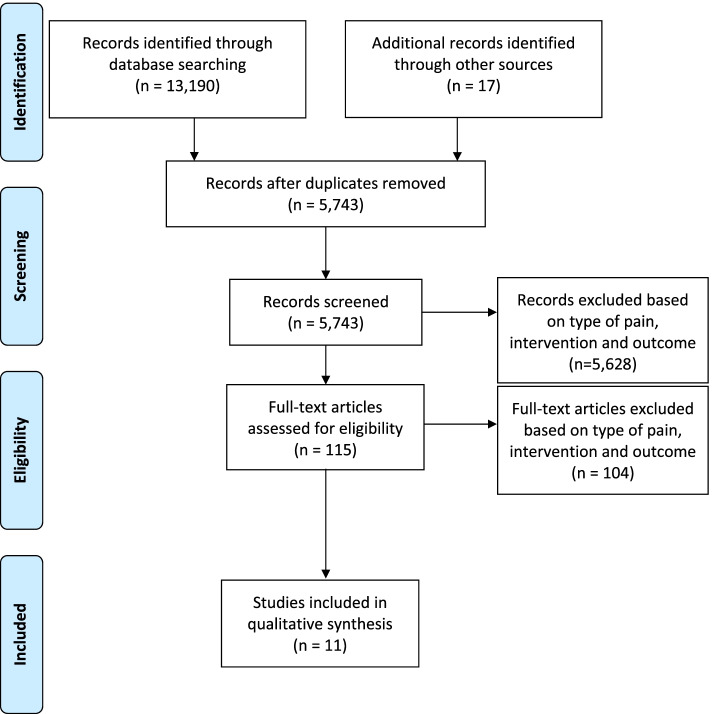
PRISMA diagram showing the flow of studies through phases of the review. Note: colour print is not required

Electronic searches identified 13,190 studies, 2,548 from OVID MEDLINE, 4,542 from OVID Embase, 2,280 from CINAHL, 2,128 from OVID EBM Reviews Cochrane Central Register of Controlled Trials, 441 from PsychINFO and 1,251 from SPORTDiscus from inception to 26th March 2021 (Fig. 1). After the screening of titles and abstracts, 115 full text papers were retrieved. On examining the full text, a further 104 studies were excluded as they either: (i) investigated a healthy cohort or a cohort without musculoskeletal pain (n= 55), (ii) did not have aerobic exercise as the only intervention or included several interventions without providing the data for aerobic exercise separately (n=9), (iii) did not measure pain sensitisation as the outcome (n=27), or (iv) had an inappropriate study type (e.g. systematic review or narrative) (n=13). No additional studies were identified from reference checking of key narrative reviews, systematic reviews, and meta-analyses, resulting in a total of 11 studies being included in the review.

### Characteristics of included studies

The characteristics of the included studies are reported in Table 2. Of the 11 included studies, seven studies had an experimental, repeated measures design [33, 37, 41, 45–48] and four were randomised controlled trials [49–52]. Five studies recruited individuals with chronic low back pain [33, 37, 47, 48, 52], three studies examined those with chronic musculoskeletal pain [33, 37, 45, 46, 51], two investigated individuals with neck pain [49, 50], and one included individuals with knee pain due to OA [41]. Four studies were conducted in Denmark [45–47, 50], two studies were undertaken in the United States of America [37, 52], and the remaining studies were conducted in Belgium [33], Ireland [41], Turkey [51], Spain [48] and Poland [49]. Three studies were published before 2010 (in 2005 [37], 2009 [50] and 2010 [33]), 5 studies were published between 2016-2018 [41, 45, 46, 49, 51], and the remaining 3 studies were published in 2020 and 2021 [47, 48, 52].

**Table 2:** Characteristics of the included studies examining the effect of aerobic exercise on pain sensitization

Author (year), Country	Demographics n (% Female) Mean(SD) age (years)	Study inclusions/exclusions of musculoskeletal (MSK) pain group	Pain sensitization assessment	Aerobic exercise protocol (including type and dosage)	Results	Conclusions
**Experimental, repeated measures studies**						
**Hoffman (2005), USA**	*Chronic LBP subjects* n: 8 (50%) Age: 40(10) *Healthy subjects* n: 10 (70%) Age: 34(8)	*Inclusions* -LBP≥1 year -Clinical pain: stable and non-neurological *Exclusions* -Use of narcotics -Inability to walk without a device -Sacroiliac joint dysfunction -Involvement in a regular exercise or treatment program -Major surgery in the past year -History of spondyloarthropathy -Spinal infection, fracture, spondylolisthesis or malignancy -Cardiac, pulmonary or metabolic disorders, or diseases involving sensory nerves -Conditions preventing safe participation in exercise -Pregnancy	Device: Pressure pain stimulator with Lucite edge (6mmx0.25mm) Force: 9.8 N Locations: dorsal surface of the middle phalanx of the non-dominant index finger Duration: 2 min Scale: 100mm VAS	-1st PPT 1 min before starting aerobic exercise -Cycling on ergometer for 5 min at 50% VO_2_max followed by 20 min at 70% VO_2_max -2nd PPT 2 min after completion of cycling -3rd PPT 28 min after completion of the 2nd PPT (32 min post exercise)	Mean (SD) pressure pain ratings were significantly lower at 2 min (57(26) mm) and 32 min post exercise (62(27) mm) compared to pre-exercise values (79(12) mm) (p<0.05).	Exercise-induced analgesia to an experimentally induced pressure pain was evident for >30 min after aerobic exercise from cycling in people with chronic low back pain and minimal/ moderate disability.
**Meeus (2010), Belgium**	*Chronic LBP* n=21 (48%) Age: 41.6(12.4) *Chronic fatigue syndrome with chronic pain* n=26 (19%) Age: 41.5(11.4) *Healthy subjects* n=31 (32%) Age: 40.0 (12.6)	*Inclusions* -18-65 years - Non-specific LBP≥3 months - Sedentary *Exclusions* -Pregnancy and ≤1 year postnatal -Neurological or cardiovascular problems - Specific LBP pathology -History of spinal fracture, spinal surgery, severe degenerative change, severe scoliosis, osteoporosis, obesity, radicular signs, malignancies and metabolic or rheumatological diseases	*Device*: Fisher algometer *Force*: Increased at a range of 1 kg/s *Locations*: skin web between thumb and index finger, 5 cm lateral to L3 spinous process, insertion of deltoid and at the proximal third of the calf *Measures*: Mean of last 2 of 3 consecutive measurements at each site separated by 10 s before and after each exercise bout.	6 bouts of exercise on a bicycle ergometer -Incremental, starting at 20 W and increasing in steps of 10 W/minute -Each bout began with a warm-up period, starting from 0 and increasing by 1 W every 2 s -Exercise consisted of 2 incremental 1-minute steps -Each bout finished with a cool down of 30 s -Subjects instructed to stop when tired or couldn’t pedal at frequency of ≥70 rpm -6th exercise bout ended at 130 W	*Baseline* There were no significant differences in PPTs between healthy subjects and patients with chronic LBP (p=NA). *Post-exercise* The mean(SD) PPTs increased following exercise in healthy and CLBP individuals: Healthy: 7.11(2.74) to 7.56(3.17) (p=0.001). CLBP: 8.10(3.02) to 8.28(3.49) (p=0.001).	After submaximal aerobic exercise, mean pain thresholds increased in patients with chronic LBP. There was no evidence of hyperalgesia and abnormal central pain processing during submaximal aerobic exercise in individuals with chronic LBP.
**Vaegter (2016), Denmark**	*Chronic musculoskeletal pain* n: 61(69%) Age: 45.4(11.2) Low (LPS; N=30) and high (HPS; N=31) pain-sensitivity groups were created on the basis of a median split of the average PPTs.	*Inclusions* -Chronic musculoskeletal pain (37 low back, 16 neck, 7 shoulder, 1 elbow) -Referral to multidisciplinary pain clinic Exclusions -Neurological, psychiatric or CVD conditions	*Device*: Manual handheld algometer *Force*: Increment rate of 30 kPa/s over stimulation area 1cm^2^ *Locations*: Middle of both quadriceps femoris, dominant biceps brachii and non-dominant upper trapezius muscle *Measures*: Before, after, and 15 min after the exercise	Aerobic stationary cycling -Age-related target HR corresponding to 50% VO_2_max and 75% VO_2_max -Patients pedaled at ~70 revolutions per minute -First 2 min: warm up (HR: 50% VO_2_max) -Resistance increased over next 3 min until HR: 75% VO_2_max -Continuation to maintain this HR for 10 min	*Baseline* PPT and pain tolerance were decreased in the HPS group compared to the LPS (P=0.001; 0.02 respectively). *Post-exercise* Widespread PPTs increased after exercise in both groups HPS: 272.8(158.0) to 319.1 (162.1) (p<0.05) LPS: 574.7(362.1) to 646.3(378.8) (p<0.05) Cuff PPT increased and pain tolerance limit decreased after exercises in LPS only (p<0.001). Temporal summation of pain was increased after bicycling in HPS (p<0.005). Pain tolerance increased after exercise in both groups (p<0.001).	Hypoalgesia after the exercise was impaired in patients with chronic pain and high pain sensitivity compared with patients with less pain sensitivity.
**Vaegter (2018), Denmark**	*Chronic musculoskeletal pain* n: 54(72%) Age: 45.7(11.2) Participants were subgrouped into high and low kinesiophobia based on the recommended threshold for a high degree of kinesiophobia on the Tampa scale.	All patients recruited after referral to a multidisciplinary pain clinic	*Device*: Manual handheld algometer *Force*: Increment rate of 30 kPa/s over stimulation area 1cm^2^ *Locations*: Middle of both quadriceps femoris, dominant biceps brachii and non-dominant upper trapezius muscle *Measures*: Before, after, and 15 min after the exercise, 2 assessments per site, average used for analysis	2 exercise conditions on 2 different days -Cycling -Isometric contraction Aerobic stationary cycling -Age-related target HR corresponding to 50% VO_2_max and 75% VO_2_max were determined -Patients pedaled at ~70 revolutions per minute -First 2 min: warm up (HR: 50% VO_2_max) -Resistance increased over next 3 min until HR: 75% VO_2_max -Continuation to maintain this HR for 10 min	*Baseline* The low kinesiophobia group had higher PPTs than the high kinesiophobia group, however no significant differences were found between the groups (p=0.09-0.59). *Post-exercise* No significant differences were found in percentage increase in PPTs between the high and low kinesiophobia groups post-exercise (p=0.12-0.58).	Although kinesiophobic beliefs influence pain intensity, they did not influence PPTs and EIH significantly, suggesting that exercise can induce hypoalgesia in subjects with chronic musculoskeletal pain, regardless of such belief.
**Fingleton (2017), Ireland**	*OA group* n:40 Divided into: -Abnormal CPM (decrease or absence of change in PPTs) (n=19) -Normal CPM (increase in PPTs) (n=21) *Control group* n:20 aged and sex matched subjects	*Inclusions* -Knee OA based on ACR criteria and pain >3/10 on a numerical rating scale -Main pain from knee OA *Exclusions* -Total knee replacement and if <90 degrees knee flexion -Rheumatologic disease such as RA, fibromyalgia or ankylosing spondylitis - Neurologic disorder such as Parkinsons disease, shingles, multiple sclerosis or stroke - Cognitive impairment - Current use of antidepressants or anticonvulsants	*Device*: Handheld pressure algometer *Force*: 2cm^2^ probe, pressure applied at a rate of 30KPa/s *Locations*: Medial joint line, quadriceps femoris muscle and volar surface of the forearm *Measures*: Average of 2 PPT measurements was recorded for each site	Aerobic exercise protocol -Cycle ergometer -Submaximal exercise protocol used: Aerobic Power Index test -Exercise duration varied between 4 and 10 min -Pain was monitored on a numerical rating scale after each minute -If pain at the knee joint exceeded 3/10, the participant’s workload was reduced by 25 W by decreasing rate of pedaling and/or resistance	There were significant differences between abnormal CPM, normal CPM and control groups for changes in PPTs during and post-aerobic exercise (F_2,55_=4.860, p=0.01) The abnormal CPM group showed a decrease in PPTs (168.9(43.1) to 152.8(52.3)), while the normal CPM and control groups showed an increase in PPTs (184.3(58.1) to 205.7(76.1) (P<0.05) and 218.0 (93.2) to 237.5 (111.7) p>0.05 pre and post exercise respectively).	Knee OA patients with abnormal CPM demonstrated significantly increased pain sensitivity in response to exercise, while knee OA patients with normal CPM and controls demonstrated a significant decrease in their pain sensitivity in response to exercise suggestive of normal function of EIH.
**Vaegter (2021), Denmark**	n: 96 (37.5%) Age: Mean (range): 47(20-73)	*Inclusions* - Individuals ≥ 18 years who were adept in Danish -Pain primarily in the lower back (+/- pain radiating to the legs) Exclusions -Pregnancy, neurological, psychological or cardiovascular diseases, and current or previous alcohol or drug addiction	*Device*: Manual pressure algometry *Force*: stimulation probe of 1 cm^2^ was used and the pressure was increased with 30 kPa/s *Locations*: Left erector spinae and left calf muscles *Measures*: average PPTs across two repetitions at each site	6 min walk test on 20 m course between 2 cones	No significant main effects were found for PPTs. However, a significant interaction between time and Walk-Pain Index was found in the RM-ANOVA of the PPTs (F(1,94)=5.56, p=0.02, partial ƞ2 = 0.056). Post hoc testing showed an increase in PPTs after walking in individuals who reported no or little increase in NRS scores of back pain intensity, and a decrease in PPTs after walking in individuals who reported an increase of 2 or more in NRS back pain intensity scores.	This study found most individuals experienced exercise-induced hypoalgesia after walking, with the exception of those that reported an increase in pain during walking and subsequently no hypoalgesia afterwards.
**Sitges (2021), Spain**	*Aerobic exercise* n: 21 (57%) Age: 42.5 (9.72*)* *Control* n:19 (58%) Age: 40.71 (9.95)	*Inclusions* -18–59 years -NSCLBP >6 weeks or with at least 3 episodes of LBP (lasting >1 week) in the year prior to the study *Exclusions* -High functional impairment compromising such activities as walking, sitting, or getting up from a chair, pain at time of evaluation and/or intervention >5 (out of 10) on the VAS, history or presence of sciatic radiating pain, referred pain, or OA of lower extremities, spine surgery, spinal or pelvic fracture, hospitalization for serious trauma, injuries, or traffic accidents, and systematic diseases affecting the locomotor system.	*Device*: Digital algometer *Force*: Maximum 5 kg/cm^2^, no further details given *Locations*: Erector spinae and gluteus medius muscles, sacrum and forefinger *Measures*: no details given.	Aerobic intervention consisted of walking on a treadmill for 20 min at low–moderate intensity (65.9%±7% of maximum heart rate and 3.02±1.04 using the Borg Scale of Perceived Exertion) No information provided on the control group.	Mixed-model ANOVA revealed significant main effects of time on subjective PPIRs (F1, 77=13.142, p=0.001, ηp^2^=0.146). Bonferroni post hoc analyses showed: lower PPIRs (2.581±1.584 vs. 2.865±1.629, *p*=0.001) and lower PPTs (2.581 ±1.584 vs. 2.0.865±1.629, p=0.001) after intervention than before. Mixed-model ANOVA revealed significant main effects of time on the pressure pain–sensitivity index (F 1, 77=7.074, p<0.001, Greenhouse-Geisser correct: ηp^2^=0.084). Bonferroni post hoc analyses showed a reduction in pain sensitivity after intervention (1.217±0.945 vs. 1.082±0.918, *p*=0.010).	This study showed reductions in pain sensitivity after an aerobic exercise intervention in patients with non-specific, chronic low back pain.
**Randomised controlled trial**						
**Öte Karaca (2017), Turkey**	*Aerobic exercise*: n: 25 (64%) Age: 43.7(10.8) *Control*: n: 25 (68%) Age: 44.9(7.9)	*Inclusions* -MSK pain > 3 months -Recruited from Physical Medicine Rehabilitation Department outpatient clinic *Exclusions* -Uncontrolled hypertension or arrhythmias -Inflammatory arthritis -Fibromyalgia -Taking analgesia -Physical therapy -Regular exercise in the last 6 months	*Device*: Mechanical pressure algometer *Force*: 1cm^2^ diameter pressure surface, 1 kg/s increments *Locations*: Midpoint of forehead, bilateral volar surface of forearm and thumb nails *Measures*: 3 consecutive measurements at 30-60 s intervals and mean taken to be pressure threshold	*Intervention* -Submaximal aerobic exercise program -Treadmill walking -30 min 5 days a week for 2 weeks -70-85% maximum HR -With conventional physical therapy *Control* -Conventional physical therapy	PPT sum increased significantly in the exercise group from 19.9(6.1) to 22.0(6.3) (p=0.02), but was unchanged in the control group (20.7(5.4) to 20.9(6.7) (p=0.9)). There was a significant increase in exercise duration in the exercise group compared with the control group (p=0.0002). Pain intensity in both groups decreased significantly after exercise (p<0.001).	Short-term aerobic exercise along with conventional physical therapy decreased pain sensitivity in individuals with musculoskeletal pain.
**Nielsen (2009), Denmark**	*Aerobic exercise*: n: 11 (100%) Age: 49(7) *Control*: n: 5 (100%) Age: 48(11)	*Inclusions* -Female office workers with monotonous, repetitive tasks -Chronic pain in the neck, doctor-verified tightness of the upper trapezius muscle (UTM) and tenderness on UTM palpation -Specific criteria 1) Pain for > 30 days in past year in neck/shoulder region, but with no more than 3 regions with symptoms 2) At least ‘‘quite a lot’’: on an ordinal scale of ‘‘a little” to ‘‘very much’’ 3) Frequent: at least once a week on an ordinal scale of ‘‘seldom” to “almost all the time’’ 4) Intensity: ≥2 on a scale from 0 to 9 *Exclusions* -Previous trauma, life-threatening diseases, whiplash injury, cardiovascular diseases, arthritis in the neck and shoulder	*Device*: Electronic pressure algometer *Force*: Contact diameter 10mm, increments of 30 kPa/s *Locations*: Descending part of the trapezius muscle and the middle of the non-painful TA muscle *Measures*: Before and after intervention, 3 times with at least 1 min between the measurements	*Intervention* -Leg bicycling on stationary Monark ergometer at 70% maximal oxygen uptake for 20 min 3 x pw for 10 weeks -Initial load 50% based on HR and gradually increased to 70% during the 10 weeks *Control* -No physical training, general health advice	*Case-control study: Baseline* PPTs were significantly lower in neck/shoulder pain group than the control group: Trapezius Neck pain: (280(82) kPa) Control: (479(119) kPa) (p< 0.05). Tibialis anterior (reference muscle): Neck pain: (302(110) kPa) Control: (464 (134)) (p< 0.05) *Intervention study Post-exercise* PPTs in tibialis anterior were increased in the cycling group [from 311 (113) to 386 (107) kPa; p<0.01].	Physical exercise, in general, lowers pain perception, resulting in normalisation of PPT in pain-free muscles.
**Kocur (2016), Poland**	*Nordic walking* n: 22 (100%) Age: 54.5(3.7) *Control* n: 22 (100%) Age: 56.7(2.9)	*Inclusions* -Females 50-60 years, sedentary work for ≥ 6 h per day for ≥10 years -Moderate or mild pain (VAS <6) in cervical area -Lack of additional physical activity in free time *Exclusions* -Locomotor system disorders preventing physical exercises -Physical work, work in standing position, sedentary work for < 10 years -Participation in physical activity over the past year -Acute inflammatory conditions -Acute pain in the cervical area and shoulders VAS >6 -Idiopathic pain -Cardiovascular or pulomonary disorders or other internal diseases	*Device*: Electronic pressure algometer *Force*: Not specified *Locations*: Trapezius par desc, mid trapezius, latissimus dorsi, infraspinatus, pectoralis major, triceps brachii, brachio-radialis *Measures*: Test performed twice 10 s apart, second result used for calculations	*Intervention* -12 week Nordic walking training: 3 times a week of 1 h -Outdoors with at least 2 instructors controlling the marching technique and regulating pace -Preceded by 10 min warm-up and ended with 5-minute cool down -Intensity between 40-70% of HRR *Control* -Told not to change their movement routines and habits for the period of 12 weeks	There was significant improvements in PPTs for Nordic Walking: Trapezius pars descendens (1.32(0.5) to 1.99(0.6) p=0.002), Middle trapezius (2.92(0.9) to 3.30(0.8) p=0.002), Infraspinatus (1.63(0.6) to 2.93(0.8) p=0.001) Latissimus dorsi (1.66(0.6) to 2.21(0.5) p=0.02) No statistically significant improvement in PPTs were observed in pectoralis major, triceps and brachioradialis in the treatment group p=(0.12-0.39). No improvement was recorded in any muscle groups in the control group (p=0.05-0.92).	Nordic Walking has a high potential of reducing sensitivity to pressure (increased PPT) in the muscles of that region.
**Bruehl (2020), United States**	*Aerobic* n: 44 (55.3%) Age: 40 (10) *Control* n: 49 (65.9%) Age: 41.9 (9.45)	*Inclusions* -18-55 years -Daily low back pain of ≥3 months duration, with an average past month pain intensity (VAS) of ≥3/10 -Medical provider diagnosis consistent with CLBP -No self-reported history of liver or kidney disorders, PTSD, BPD, psychotic disorder, diabetes, seizure disorder, alcohol or drug dependence, or daily use of opioid analgesics -Engaged in moderate or vigorous exercise <2 days per week and <60 min per week *Exclusions* -Self-reporting CP related to malignancy or autoimmune disorders -Pregnancy	*Device*: Computerised Medoc TSAII NeuroSensory Analyser *Force*: 40 °C and increasing at a ramp rate of 0.5 °C per second until tolerance was reached *Locations*: slightly different location of the ventral forearm for each stimulus to avoid local sensitization effects *Measures*: 3 trials were conducted for heat pain tolerance (with the mean value used for analyses)	*Intervention* -Supervised, individual aerobic exercise training program 3 times per week for 6 weeks -Exercise session included a 5-minute warm-up, 30 min of aerobic exercise and a 5-minute cool-down -Treadmill walking/running, stepping, elliptical, or cycling exercise as preferred by the participant -Duration and intensity of exercise was progressively increased -Participants began with 10-15 min of exercise at 40-55% HRR (RPE =11-12, light) during the first week, 20-30 min of exercise at 55-70% HRR (RPE = 12-13, somewhat hard) during the second week, then 30 min of exercise at 70-85% HRR (RPE=14-16, hard) for the remainder of the study *Control* -Asked to maintain their normal daily activity levels throughout the study	*Baseline* Evoked thermal pain responses at baseline did not differ significantly between groups. *Post-exercise* -Significant main effect of intervention Group on MPQ-SF Total ratings [F(1,77) =5.80, P =0.018, h2 = 0.064] -Participants in the exercise group displayed slightly improved pain responsiveness (decreased thermal pain ratings) after the 6-week intervention (M = 0.33, SE =0.70), whereas control group participants reported an increase in thermal pain responses over time (M=22.04, SE= 0.68) -However, group-level mean reduction in evoked pain responsiveness observed in the exercise condition was not significantly different from zero [t(37) 5 0.63, P = 0.54] -Women in the exercise group exhibited significantly larger increases in EO function (M = 1.68, SE = 0.91) than women in the control group [M = -0.93, SE =0.80; F(1,46) = 5.35, P =0.025]. This intervention effect was not significant in men.	Supervised progressive aerobic exercise training can significantly decrease pain in individuals with CLBP, with evidence supporting enhanced pain inhibitory function.

MSK -musculoskeletal, LBP -low back pain, VAS -visual analogue scale, VO_2_ -volume of oxygen consumed, PPT -pressure pain threshold, HPS -high pain sensitivity, LPS -low pain sensitivity, HR -heart rate, HRR -heart rate reserve, EIH -exercise-induced hypoalgesia, OA -osteoarthritis, CPM -conditioned pain modulation, ACR -American College of Rheumatology, UTM -upper trapezius muscle, kPa -kilopascals, NA – Not available. PTSD -posttraumatic stress disorder, BPD -bipolar disorder, NSCLBP – non-specific chronic low back pain, PPIRs - pain pressure intensity ratings, NRS -numerical rating scale, MPQ-SF -McGill Pain Questionnaire (short form), RPE -rating of perceived exertion, EO -endogenous opioids, CLBP -chronic low back pain

### Characteristics of participants

The studies ranged from having 8-96 participants. Participants had a mean age ranging from 34 to 56 years, except for the study on knee OA which did not specify the mean age of the cohort [41]. Overall 38-100% of the participants were female. However, one study did not report the number of males and females [41].

### Definitions of musculoskeletal pain

A variety of definitions of musculoskeletal pain were used in the 11 studies. Of the 5 low back studies, two studies defined low back pain as non-specific pain lasting longer than three months [33, 52], and the other studies used definitions such as clinically stable, non-neurological pain lasting longer than one year [37], pain in the lower back with or without pain in the lower limbs [47], and pain lasting > 6 weeks or at least 3 episodes (>1 week) in the last year [48]. In the 2 studies of chronic musculoskeletal pain, more than half of the cohort experienced low back pain, and the remainder reported neck, shoulder and elbow pain, with the duration of pain not specified [45, 46]. Ote Karaca et al. recruited individuals who had regional musculoskeletal pain exceeding three months in duration [51]. Neilsen et al. recruited participants who had neck or shoulder pain lasting for more than 30 days [50], while Kocur et al. recruited participants who had cervical pain with the exclusion of “acute pain in the cervical area and shoulders” [49]. In the study by Fingelton et al. which examined individuals with knee OA, OA was diagnosed based on the American College of Rheumatology classification, and needed to be the main source of pain, and exceed 3 on an 11 point numerical rating scale [41].

Four of the included studies classified participants into subgroups based on pain sensitivity, kinesiophobia, self-reported pain intensity during activity, and conditioned pain modulation [41, 45–47]. Vaegter et al. (2016) examined high and low pain sensitivity subgroupsbased on a median split of the average PPTs for men and women [45], while their later study in 2018 examined high and low kinesiophobia subgroups, where the subgroups were created based on the recommended threshold for a high degree of kinesiophobia using the Tampa Scale of Kinesiophobia (score≥38) [46]. In addition, the most recent study by Vaegter et al. examined change in pain sensitisation in individuals that reported an increase (≥2/10) in their back pain during walking compared to those that reported no or limited increase (<2/10) [47]. The study of OA classified participants as “abnormal conditioned pain modulation (CPM)” where there was a decrease in PPTs or absence of change, or “normal CPM” where there was an increase in PPTs [41].

### Aerobic exercise prescription

Five experimental, repeated measures studies and one of the randomised controlled trials used cycle ergometry as the prescribed aerobic exercise [33, 37, 41, 45, 46, 50], while one repeated measures study and randomised controlled trial used treadmill walking [48, 51] and the remaining 3 studies used Nordic walking [49], a 6 min walk test [47], and selection from a range of aerobic activities, including walking, stepping, elliptical exercise and cycling, as per the participant’s preference [49, 52].

The degree of exertion was standardised in two ways. The first involved calculating target VO_2_ maximums through maximal or submaximal exercise tests prior to the prescribed aerobic exercise, while the second was based on target age-related heart rates [37, 41, 45, 46, 48–52]. Two studies did not standardise the degree of exertion [33, 47].

In the 7 repeated measures studies, the exercise protocols were submaximal, incremental and completed in a single setting over 4 to 30 min, ranging from: (i) commencing at 50% maximal VO_2_ for 5 min and increasing to 70% maximal VO_2_ for 20 min as a single bout [37], (ii) starting at 20 W after a short warm-up period and increasing by 10 W per minute for 2 min per exercise bout followed by a 30 s cooling down period for 6 exercise bouts [33], (iii) starting at a heart rate corresponding to 50% maximal VO_2_ for 2 min, increasing to 75% over 3 min and continuation at that heart rate for a further 10 min in a single bout [45, 46], (iv) starting at 25 W and increasing by 25 W per minute to 75% age-predicted maximal heart rate for a duration of 4-10 min [41] and (v) increasing intensity to a low-moderate intensity of 70% of maximum heart rate [48]. One study did not have a specific exercise protocol but instructed participants to walk as far as possible within the 6 min time frame [47].

The randomised controlled trials described a more prolonged submaximal aerobic exercise protocol. Ote Karaca et al. described treadmill-based aerobic exercise at an intensity of 70-85% age-related target heart rate for 30 min five days a week for two weeks [51]. Neilsen et al. described a stationary, ergometer-based aerobic exercise at 50% initial load based on heart rate, gradually increasing to 70% during the 10 weeks [50]. Kocur et al. described Nordic walking between 40 and 70% heart rate reserve for 1 h, 3 times a week for 12 weeks with each session preceded by a warm up and followed by a cool down phase [49]. Bruehl et al. started participants with 10-15 min of exercise at 40-55% heart rate reserve during the first week, followed by 20-30 min at a 55-70% heart rate reserve during the second week, and then 30 min at a 70-85% heart rate reserve for the remainder of the study [52].

### Assessment of pain sensitisation

The outcome we assessed was pain sensitisation, which was measured with pressure pain testing in 10 studies and thermal pain testing in one study. Pressure pain testing involved producing pressure over a small surface area, participants identifying when a pressure sensation was first perceived as pain and the corresponding pressure or the pain level being recorded. Sites of pressure pain testing were variable [33, 37], with the quadriceps femoris [41, 45, 46], and [45, 46, 51]trapezius [45, 46, 49, 50], most commonly used [49]. In most of the studies, pressure applied was incremented either at a rate of 1 kg/s or 30 kPa/s over 1cm^2^ or 2cm^2 ^[33, 37, 41, 45, 46, 49–51]. In contrast, the trial by Bruehl performed thermal pain testing which involved applying a thermode to the skin of the forearm starting at 40 °C and increasing at a ramp rate of 0.5 °C per second until tolerance was reached, at which the temperature was recorded and the participant rated their pain intensity [52].

#### Risk of bias

The studies in this review were largely of low to moderate risk of bias, with two studies having low risk of bias [45, 46], six studies having moderate bias [33, 37, 41, 47–49], and three studies having high risk of bias [50–52]. All seven observational studies adequately assessed both the exposures and outcomes and ensured that the co-interventions were similar between both cohorts. However, four studies did not match exposed and unexposed for all variables that are associated with the outcome of interest or adjust for these prognostic variables [33, 37, 47, 48], and 3 of the studies did not draw cohorts from the same population [33, 37, 41]. One of the 4 randomised controlled trials reflected a moderate risk of bias [49], while the other three reflected a high risk of bias as they had no allocation concealment and did not mention blinding of participants or personnel [50–52].

### Clinical heterogeneity

Based on the Clinical Diversity In Meta-analyses (CDIM) tool[44], we calculated a score of 17 (of a total score of 22) for the clinical trials included in this review, indicating high clinical heterogeneity. In particular, scores of 2 were evident in 3 of the 4 domains, including the population, intervention and outcome domains, reflecting significant heterogeneity. Similarly, when examining the observational studies we found significant clinical heterogeneity, which was evident across all domains.

### Effect of aerobic exercise on pain sensitisation

#### Question 1: Does aerobic exercise effect pain sensitisation? If so, what effect does it have?

All 11 studies reported a hypoalgesic effect of aerobic exercise on pain sensitisation in participants with chronic musculoskeletal pain. A summary of these results is presented in Tables 2 and 3. Moreover, when we examined the risk of bias of the included studies and only considered the 8 studies that had low to moderate risk of bias, our findings of a reduction in pain sensitisation following aerobic exercise, remained unchanged. However, there was evidence from two studies that examined specific musculoskeletal pain subgroups, including those with abnormal conditioned pain modulation or an increase in low back or leg pain while walking, that not all subgroups experienced a reduction in pain sensitisation.

**Table 3:** Summary of the prescribed aerobic exercise and percentage improvement in pain sensitization across the studies

Author (Year)	Type	Duration	Frequency	Intensity	Number of time points	Endpoint measured (units)	Pre-exercise Mean (SD) PPTs/MPRs^a^	Post-exercise Mean (SD) PPTs/MPRs/MPQ score^a^	Percentage change in PPTs/MPRs/MPQ score^a^
**Chronic musculoskeletal pain**									
Randomised controlled trials									
Ote Karaca (2017)	Treadmill	30 min	5 x per week for 2 weeks	Submaximal – 70-85% maximum HR	2 –before and after exercise	PPT sum (kg/cm^2^)	19.9 (6.1)	22.0 (6.3) p=0.023	10.6% increase in mean PPTs
							20.7 (5.4)	20.9 (6.7) p=0.898	
Repeated measures studies									
Vaegter (2016)	Cycle ergometry	15 min	1 session	Submaximal -50% and 75% VO_2_max	3 –before, immediately after and 15 min after exercise	Widespread PPTs (kPa)	High pain sensitivity 272.8 (158.0) Low pain sensitivity 574.7 (362.1)	High pain sensitivity 319.1 (162.1) 290.8 (158.5) p<0.05 Low pain sensitivity 646.3 (378.8) 563.5 (343.1) p<0.05	High pain sensitivity 17.0% increase in mean PPTs Low pain sensitivity 12.5% increase in mean PPTs Overall mean 14.8% increase in mean PPTs
Vaegter (2018)	Cycle ergometry	15 min	1 session	Submaximal -50% and 75% VO_2_max	3 –before, immediately after and 15 min after exercise	PPTs	No mean PPT values given	No mean PPT values given	No mean data provided
							No mean PPT values given	No mean PPT values given	
**Chronic low back pain**									
Randomised controlled trials									
Bruehl (2020)	Aerobic exercise - treadmill walking/running, stepping, elliptical, or cycling exercise as preferred by the participant	30 min with 5 min warm up and cool down	3 sessions per week for 6 weeks	Submaximal- 70% and 85% HRR (RPE=14-16, hard)	2 -Before and within 10 days of final exercise session	MPQ-SF (Total)	Pre: 10.31(9.29)	Post: 9.91(8.67)	Exercise group 3.9% decrease in the MPQ-total pain measure Control group 27.1% increase in the MPQ-total pain measure
Repeated measures studies									
Meeus (2010)	Cycle ergometry	6 bouts of incremental exercise –warmup and 60 s exercise phase	1 session	Submaximal -20 W and increasing in steps of 10 W/minute	2 –before and immediately after exercise	Mean PPTs (kg/cm^3^)	8.1 (3.02)	8.28 (3.49) p=0.001	2.2% increase in mean PPTs
Hoffman (2005)	Cycle ergometry	25 min	1 session	Submaximal – 50-70% VO_2_	3 –before, immediately after and 32 min after exercise	Mean pressure pain ratings (100mm VAS)	79 (12)	57 (26) p<0.05 62 (27) p<0.05	27.8% decrease in MPRs
Vaegter (2021)	6-minute walk test	6 min	1 session	Submaximal	Immediately before and after	PPTs	Walking pain index <2: Lower back 586 (149-1665) Walking pain index ≥2: Lower back 450 (203–1,368)	No PPT values given	Walking pain index <2: Absolute change in PPT Lower back (kPa): 38 (153) 6.6% increase Walking pain index ≥2: Absolute change in PPT Lower back (kPa): -17 (112) 3.8% decrease
Sitges (2021)	Treadmill walking	20 min	1 session	Low–moderate intensity (65.85%±7% of maximum heart rate and 3.02±1.04 using the Borg Scale of Perceived Exertion)	Before and after the exercise session, no further details given	PPTs	Gluteus medius PPTs 2.66 (1.02)	Gluteus medius PPTs 2.81 (0.97)	Exercise group 0.16 (0.55) 6.0% increase Control group 0.12 (0.39) 4.4% increase
							Gluteus medius PPTs 2.97 (1.20)	Gluteus medius PPTs 3.10 (1.24)	
**Neck pain**									
Randomised controlled trials									
Neilsen (2010)	Cycle ergometry	20 min	3 x per week for 10 weeks	Submaximal -50-70% maximum HR	2 –before and after exercise	Tibialis anterior and trapezius PPTs (kPa)	311 (113) Trapezius -no significant increase	386 (107) p<0.01 Trapezius -no significant increase	24.1% increase in mean PPTs
Kocur (2017)	Nordic walking	1 h	3 x per week for 12 weeks	Submaximal -40-70% HRR	2 –before and 1-2 days after last NW training session	PPTs -descending trapezius, mid trapezius, lat dorsi, infraspinatus, pec major, triceps brachii and brachioradialis (kg/cm^2^)	Descending trapezius -1.32 (0.5) Infraspinatus -1.63 (0.6) Lat dorsi -1.66 (0.6) Mid trap -2.92 (0.9) No significant increase -pecmajor, triceps brachii, brachioradialis	Descending trapezius -1.99 (0.6) p=0.002 Infraspinatus -2.93 (0.8) p=0.001 Lat dorsi -2.21 (0.5) p=0.02 Mid trap -3.3 (0.8) p=0.002 No significant increase -pec major, triceps brachii, brachioradialis	No mean data provided
**Osteoarthritis**									
Repeated measures studies									
Fingleton (2017)	Cycle ergometry	4-10 min	1 session	Submaximal - If pain at the knee joint exceeded 3/10, the participant’s workload was reduced by 25 W	2 Before and immediately after exercise	Average PPTs of knee and forearm (kPa)	Normal CPM 184.34 (58.11) Abnormal CPM 168.87 (43.03)	Normal CPM 205.73 (76.07) p<0.05 Abnormal CPM 152.75 (52.31) p>0.05	11.6% increase in mean PPTs

PPTs –pressure pain thresholds, MPRs – mean pain ratings. ^a^Where the pre and post PPTs/MPRs are split into 2 rows, the top row denotes mean pre and post PPTs in the intervention group, while the bottom row denotes mean pre and post PPTs in the control group. CPM -conditioned pain modulation; HPS –high pain sensitivity group; LPS -low kinesiophobia group; High K -high kinesiophobia group; Low K -low kinesiophobia group; HRR -heart rate reserve, VAS -visual analogue scale, MPQ(SF)Total -McGill Pain Questionnaire (Short form) total score

The five studies investigating low back pain reported an improvement in pain sensitisation after performing aerobic exercise. The study by Hoffman et al. reported a statistically significant decrease in pressure pain at two minutes and 32 min after aerobic exercise on a cycle ergometer [37]. Meeus et al. reported a similar result with an increase in mean PPTs immediately following aerobic exercise [33], as did the study by Sitges et al. which reported lower pain sensitivity and pressure pain–intensity ratings following 20 min of treadmill walking [48]. The study by Vaegter et al. reported an increase in PPTs after walking in individuals who reported no or little increase in pain while walking [47], while the clinical trial by Bruehl et al. reported larger reductions in evoked pain responsiveness in those who undertook a 6 week program involving their preferred aerobic exercise compared to controls [52].

The three studies of chronic musculoskeletal pain, including the randomised controlled trial and the two repeated measures studies, reported a statistically significant increase in pain tolerance after aerobic exercise [45, 46, 51].

The two studies examining chronic cervical pain reported increased PPTs at specific sites, although they did not report an overall mean or summation score [49, 50]. Neilsen et al. reported increased PPTs at the tibialis anterior site without significant increase in the descending trapezius, the other site measured [50]. Kocur et al. reported significant increases in PPTs in 4 of 7 of the sites measured [49]. In the control groups of both of these studies, no increases in PPTs at any of the sites was observed [49, 50].

In the study by Fingleton et al., mean PPTs were significantly increased post exercise among participants with knee OA with a normal conditioned pain modulation, but not among participants with abnormal conditioned pain modulation [41]. Vaegter et al. found increased widespread PPTs in both high and low pain sensitivity groups with effect sizes of 0.77 and 0.52 respectively, but concluded that exercise-induced hypoalgesia was slightly impaired in patients with high pain sensitivity compared with patients with low sensitivity [45].

Of the 11 included studies, 7 studies reported mean PPTs and 2 studies reported mean pain ratings pre and post exercise allowing the percentage improvement in pain sensitisation following aerobic exercise to be determined (Table 3) [33, 41, 45, 46, 48, 50, 51]. Two studies did not report mean pre and post PPTs or pain ratings. Based on the mean change in PPTs, we calculated a median (minimum, maximum) percentage improvement in pain sensitisation of 10.6% (2.2%, 24.1%).

#### Question 2: What type and dosage of exercise, including duration, intensity and frequency of exercise, was required to achieve an effect?

All of the 11 included studies reported a hypoalgesic effect when they examined aerobic exercise that involved:

1. Walking, cycling or the participants’ preferred aerobic exercise (6 studies examined cycling [33, 37, 41, 45, 46, 50], 4 examined walking [47–49, 51] and one study investigated treadmill walking/running, stepping, elliptical, or cycling [52]).
2. Submaximal exercise (9 of the 11 studies were objectively submaximal based on percentage VO_2_ or target HR (50-75% VO_2_, [37, 45, 46], 40-85% heart rate reserve [49, 52], or 66-85% maximal heart rate [33, 41, 47–52].
3. exercise of 4-60 min in duration (with 2 studies having varying exercise times depending on participants’ tolerance [33, 41]),
4. a single bout (7 of the 11 studies) [33, 37, 41, 45–48], with 4 studies examining interventions up to 5 times a week and up to 12 weeks [49–52].
5. mostly incremental (at least 7 of the 11 studies) [33, 37, 41, 45, 46, 50, 52].

It would therefore seem that a single session of submaximal, incremental exercise involving walking or cycling of at least 4 min duration based on 50-75% VO_2_, 40-85% heart rate reserve [49, 52], or 66-85% maximal heart rate [41, 48, 50, 51] may achieve a hypoalgesic effect immediately post exercise.

#### Was there an improvement in PPTs over both shorter and longer time periods?

While the majority of the repeated measures studies examined pain sensitisation before and immediately after aerobic exercise, two studies measured pain sensitisation a period of time after exercise [37, 45]. Hoffman et al. reported pressure pain ratings that continued to be lower compared with pre-exercise at 32 min post-exercise [37], while Vaegter et al. reported PPTs that were no different with pre-exercise at 15 min post-exercise [45, 46] The study by Hoffman et al. that had a hypoalgesic effect at 32 min post exercise prescribed aerobic exercise for a 25 min period, while the study by Vaegter et al. that lost the hypoalgesic effect at 15 min post exercise had a 15 min aerobic exercise prescription [37, 45]

In contrast to the repeated measures studies, the four randomised controlled trials assessed pain sensitisation after 2, 6, 10 and 12 weeks of an aerobic exercise program. Ote Karaca et al. reported a statistically significant increase in sum of PPTs after a 2 week intervention (with 5 of 7 components of the sum having individually statistically significant increases) [51]. Neilsen et al. reported increased PPTs at one of two tested sites (tibialis anterior site but not the trapezius) after 10 weeks of aerobic exercise, and Kocur et al. reported increased PPTs in four of the seven sites after 12 weeks of aerobic exercise. However, these 2 studies did not report a mean or comparison of the widespread or collective PPTs across the sites tested [49, 50]. Moreover, Bruehl et al. reported an decrease in pain sensitivity based on the MPQ-total pain measure in response to a thermal pain stimulus at 6 weeks following an aerobic exercise program [33, 37, 41, 45–52].

## Discussion

This systematic review provides evidence to suggest that aerobic exercise reduces pain sensitisation in individuals with musculoskeletal pain. All 11 studies concluded that aerobic exercise produced a hypoalgesic effect in their musculoskeletal pain cohort, with median (minimum, maximum) improvement in pain sensitisation of 10.6% (2.2%, 24.1%) post exercise. Moreover, the studies showed that aerobic exercise, involving walking or cycling, at a submaximal intensity of 50-75% maximal VO_2_ or 40-85% maximal heart rate/heart rate reserve and with incremental increases, for a 4-60 min duration, and for a single session or 1-5 sessions per week for up to 12 weeks resulted in a hypoalgesic effect. However, there was evidence from studies of specific patient subgroups, including individuals with abnormal conditioned pain modulation or an increase in low back or leg pain while walking, that performing aerobic exercise may not reduce pain sensitization in all individuals with musculoskeletal pain. Given the lack of effective treatments for pain sensitisation [11, 12], the escalating use of off-label antidepressants [53, 54], and the alarming use and side-effects associated with opioids [13–17], these findings highlight the need for further investigation to determine whether aerobic exercise may be a safe and effective, non-pharmacological “pill” for the treatment of pain sensitisation in individuals with musculoskeletal pain.

Of the 11 studies included in this review, all reported that aerobic exercise resulted in hypoalgesia in individuals with musculoskeletal pain. This has not previously been shown. A 2012 meta-analysis by Naugle et al [27] investigated the effects of aerobic exercise on pain sensitisation in both healthy adults and those with chronic pain. They concluded that there was a trend for a beneficial effect for individuals with chronic pain, but the results were highly variable, particularly with regards to the magnitude and direction of the effect sizes [27]. It was proposed that this may be explained by 3 of the 5 studies included in the meta-analysis investigating individuals with fibromyalgia or chronic fatigue syndrome, which differed in their response to aerobic exercise and demonstrated a hyperalgesic response.

**Fig. 2:**
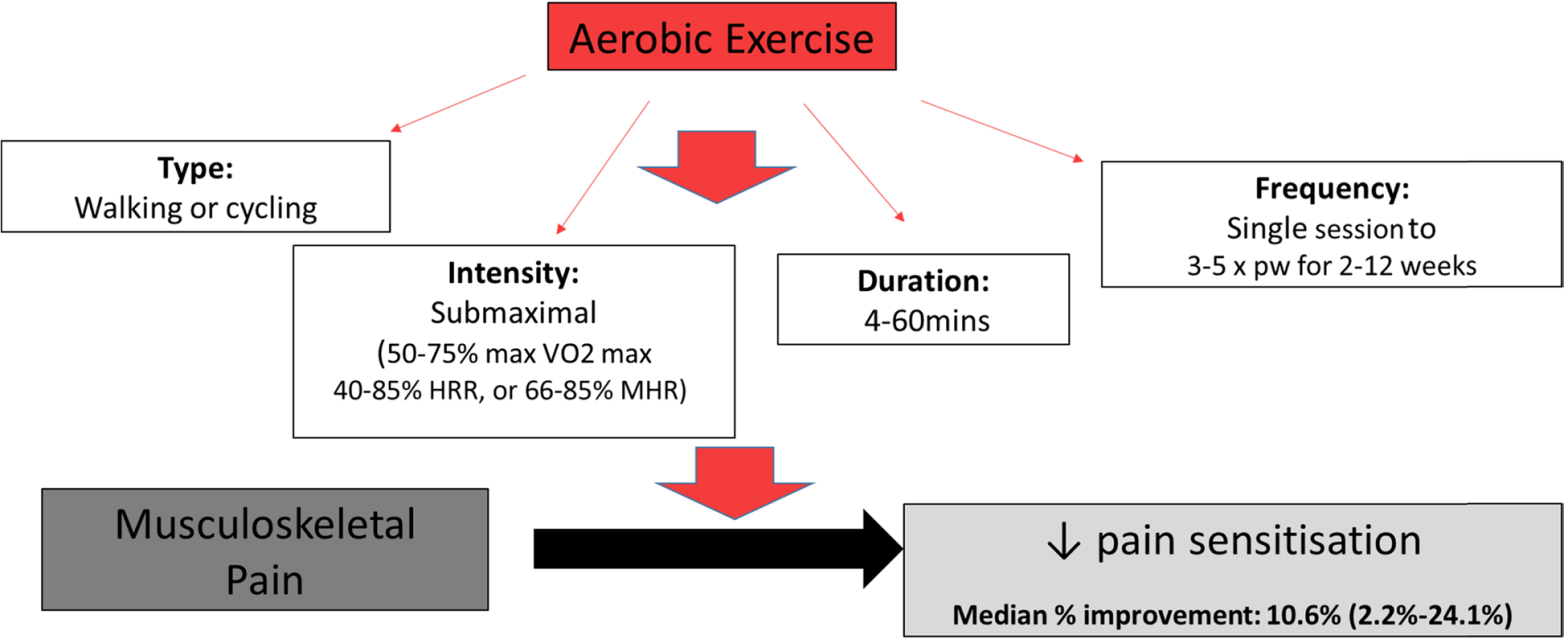
Schematic diagram showing the effect of aerobic exercise on pain sensitisation for musculoskeletal pain. Note: colour print is not required

This systematic review provides preliminary data to suggest the type and dosage of aerobic exercise which might be required to achieve a reduction in pain sensitisation. Among the studies in this review, there were a range of exercise parameters utilised in relation to frequency, intensity and duration. Overall, the studies found submaximal, incremental exercise that involved walking or cycling for at least 4 min duration based on 50-75% maximal VO_2_, 40-85% heart rate reserve, or 66-85% maximal heart rate on one or multiple occasions is needed to achieve a reduction in pain sensitisation. While the repeated measures studies prescribed a single bout of exercise, the clinical trials examined effects of 2-12 weeks of aerobic exercise and showed that there was an improvement in pain sensitisation both immediately and over periods of 2, 6, 10 and 12 weeks. Figure 2 provides a schematic representation of the effect of aerobic exercise on pain sensitisation, summarising the key features of aerobic exercise that were found to produce a hypoalgesic effect. These data are important as they have the potential to guide the selection of exercise parameters for future clinical trials examining the effectiveness of aerobic exercise to improve pain sensitivity. However, a broader approach examining other key outcomes, such as disability and health-related quality of life, will also be important. The randomised controlled trial by Ote Karaca et al. was the only trial to perform testing of PPTs and patient surveys for health-related quality of life measures before and after the intervention, with the SF-36 revealing that the aerobic exercise group benefited with improved general health perceptions and reduced role limitations because of physical problems compared with the control group [51].

Three observational studies examined whether subgroups of individuals with musculoskeletal pain differ in their pain sensitivity response to aerobic exercise. Vaegter et al. 2016 found that although individuals with high pain sensitivity experienced increased PPTs post exercise, there was a slight impairment of their exercise-induced hypoalgesia compared to those with low pain sensitivity [45]. In contrast, Fingleton et al. found that individuals with knee OA and abnormal conditioned pain modulation demonstrated increased pain sensitivity compared to those with normal conditioned pain modulation and healthy controls, and Vaegter et al. 2021 found that individuals who reported an increase in lower back or leg pain with walking had impaired exercise induced hypoalgesia compared those individuals with no increased pain with walking [45]. It is worth noting that while these subgroups have been designed as surrogates for an altered pain response at baseline, these studies defined them differently. These findings demonstrate that not all individuals with chronic pain have the same response to the prescribed aerobic exercise. We postulate that individuals with a higher pain sensitivity or abnormal conditioned pain modulation at baseline might be an inherently different subgroup of patients with chronic pain, and may exhibit similar responses to aerobic exercise as patients with fibromyalgia [55, 56]. Thus, specific patient subgroups among those with chronic musculoskeletal pain need to be considered, and potentially each assessed and prescribed exercise in a specific way to ensure benefit from such an approach.

Several mechanisms have been proposed to explain how aerobic exercise may reduce pain sensitivity. While it has been suggested that aerobic exercise may release endogenous opioids and beta-endorphins that result in hypoalgesia, it has also been proposed that it may activate descending nociceptive inhibitory mechanisms that reduce pain sensitivity [24–26, 57, 58]. Moreover, the conditioned pain modulation theory hypothesizes that descending pain inhibitory responses are challenged during a painful conditioning stimulus via opioid and non-opioid pathways [58]. For instance, lower pain scores during exercise might be reported in the presence of placing a hand in ice water (the conditioning stimulus) [58]. The evidence from this review, which indicates that aerobic exercise reduces pain sensitisation in musculoskeletal pain, has the potential to have clinical implications. This is because, current clinical guidelines recommend the use of physical activity for musculoskeletal pain, but do not suggest a particular type of exercise, nor specifically recommend aerobic exercise. For instance, the NICE guidelines for non-specific low back pain recommend the provision of a group exercise program, but state an inability to recommend aerobic exercise alone due to the uncertainty around the effect size, clinical importance of the comparisons supporting aerobic exercise, as well as lack of an economic evaluation for an individual versus group intervention [59]. Thus, clinical trials examining the efficacy of aerobic exercise for specific patient subgroups, in particular those with pain sensitisation, and that involve large sample sizes and an economic evaluation, are urgently needed. These trials have the potential to inform clinical practice and treatment guidelines surrounding exercise recommendations for the future management of musculoskeletal pain, in particular, low back pain.

This review has several strengths and limitations. The key strengths included performing systematic searches of 6 electronic databases and reference lists of key systematic reviews, summarising and tabulating data from the included studies, performing a risk of bias assessment based on Cochrane methodology, and conducting a qualitative analysis, based on study design and quality. This review was limited by the modest number of heterogeneous studies, which prevented a meta-analysis being performed. However, we were able to report a decrease in pain sensitisation across all studies irrespective of study quality and a median improvement of 10% or more in pain sensitivity in studies which provided the appropriate data. While there is no established minimal clinically important difference (MCID) for pain sensitisation, there is evidence from a recent meta-analysis that patients who experience chronic pain have a significantly lower pressure pain threshold than healthy controls [60]. The pooled pressure pain threshold mean difference was reported as 1.17 (95% confidence interval = -1.45 to -0.90) kg/cm^2^, representing a 10% difference in PPTs between healthy individuals and those with chronic pain, and may indicate that a similar change of 10.6% in PPTs in individuals with chronic musculoskeletal pain following aerobic exercise is of clinical significance. We excluded non-English studies, which may have introduced bias into the review. Although unlikely, it may be that the effect of aerobic exercise on pain sensitisation differs across populations. Moreover, while our risk of bias assessment based on Cochrane methodology reported that the studies were mainly of modest quality, this review will inform future research, in particular the need for high quality clinical trials, longer follow up periods, and a greater focus on global outcomes, including disability and quality of life.

## Conclusions

This systematic review provides evidence to suggest that aerobic exercise reduces pain sensitisation in individuals with musculoskeletal pain. The findings suggest that aerobic exercise involving walking or cycling, performed at a submaximal intensity but with incremental increases, for a 4-60 min duration and for up to 12 weeks can produce a median (minimum, maximum) percentage improvement of 10.6%(2.2%, 24.1%) in pain sensitisation. These findings support the need for further work to determine whether the effect of aerobic exercise on pain sensitisation in individuals with musculoskeletal pain also translates to improved clinical outcomes.

## Supplementary Information

**Additional file 1.**

## Data Availability

All data generated or analysed during this study are included in this published article [and its supplementary information files].

## Abbreviations

OA: osteoarthritis
PPT: pressure pain thresholds
TPT: thermal pain thresholds
