# Supplementary material for: Does aerobic exercise effect pain sensitisation in individuals with musculoskeletal pain? A systematic review
Source: BMC Musculoskelet Disord. 2022 Feb 3;23:113. doi: 10.1186/s12891-022-05047-9 (PMC8815215; doi:10.1186/s12891-022-05047-9)
Supplement: Supplementary file 1 — Additional file 1. [file 12891_2022_5047_MOESM1_ESM.docx]

**SUPPLEMENTARY FILE 1**

**OVID MEDLINE Search Strategy**

| **#** | **Searches** |
| --- | --- |
| 1 | Musculoskeletal Pain/ or Myalgia/ or Myofascial Pain Syndromes/ or Arthralgia/ or Shoulder Pain/ or Back Pain/ or Low Back Pain/ or Flank Pain/ or Metatarsalgia/ or Neck Pain/ or Arthritis/ or Osteoarthritis/ or Osteoarthritis, Hip/ or Osteoarthritis, Knee/ or Osteoarthritis, Spine/ or Patellofemoral Pain Syndrome/ or Fibromyalgia/ or Whiplash Injuries/ |
| 2 | (Musculoskeletal Pain or Myalgia or Myofascial Pain or Arthralgia or Shoulder Pain or Back Pain or Low Back Pain or Flank Pain or Metatarsalgia or Neck Pain or Arthritis or Osteoarthritis or Patellofemoral Pain or Fibromyalgia).mp. |
| 3 | (backache or backpain or vertebrogenic pain or lumbago or neckache or neckpain or polyarthritides or polyarthritis or osteoarthritides or osteoarthros#s or coxarthros#s or periarthritis or periarthritides or footache or kneepain or footpain or legpain or limbpain or armpain or musculoskeletal chest pain or lumbal syndrome or lumbalgesia or lumbar spine syndrome or lumbar syndrome or lumbosacroiliac strain or pelvic girdle pain or fibrofasc?itis or shoulder-hand syndrome or hand-shoulder syndrome or neck-shoulder syndrome or cervicobrachial syndrome or shoulder-arm syndrome or arm-shoulder syndrome or whiplash or articular pain or periarticular pain or oligoarticular pain or polyarticular pain).mp. |
| 4 | (arthralgia or anthralgia or arthrialgia or brachialgia or cervicalgia or cervicobrachialgia or chondralgia or cruralgia or chiralgia or coxalgia or coccyalgia or coccygalgia or costalgia or dactylalgia or dorsalgia or encephalalgia or epicondylalgia or gonalgia or lumbalgia or myalgia or myosalgia or melalgia or meralgia or metatarsalgia or notalgia or ostalgia or ostealgia or omalgia or patellalgia or podalgia or polymyalgia or plantalgia or polyarthralgia or pygalgia or rachialgia or rhachialgia or radiculalgia or sacralgia or sacrocoxalgia or scapulalgia or spondylalgia or spinalgia or talalgia or tarsalgia or tenalgia or thoracalgia or tibialgia).mp. |
| 5 | (arthrodynia or calcaneodynia or calcodynia or cephalodynia or cervicodynia or coccygodynia or coccydynia or coccyodynia or coxodynia or chondrodynia or dactylodynia or dorsodynia or lumbodynia or myodynia or osteodynia or pleurodynia or pododynia or rachiodynia or sacrodynia or scapulodynia or spondylodynia or tenodynia or tenontodynia or thoracodynia).mp. |
| 6 | Chronic Pain/ or Nociceptive Pain/ |
| 7 | ((chronic or nociceptive or nociplastic or somatic or persistent or longterm or long-term or widespread or wide-spread) adj pain).mp. |
| 8 | ((muscle* or muscular or musculo*) adj1 (pain* or ache* or aching)).mp. |
| 9 | ((Body or trunk or torso or limb* or extremet*) adj (pain* or ache*)).mp. |
| 10 | ((painful or aching) adj (trunk or torso or limb* or extremet*)).mp. |
| 11 | ((neck or cervical or sternum or sternal or substernal or costal or rib*1 or spine or spinal or vertebral or back or midback or dorsum or dorsal or lumbus or lumbar or sacrum or sacral or buttock* or gluteus or gluteal or sacroiliac* or sacro-iliac* or pelvic girdle or iliosacral or il#o-sacral or sacrococcygeal or sacro-coccygeal or tailbone or coccyx or coccygeal or iliac crest or iliolumbar or il#o-lumbar or piriformis or cervicobrachial or cervico-brachial or cervicogenic or discogenic or atlanto-axial or atlantodental or atlantoaxial or atlantooccipital or atlanto-occipital or temporomandibular or temporo-mandibular or mandibular or jaw or craniomandibular or cranio-mandibular or mandibulotemporal or mandibulo-temporal) adj (pain* or ache*)).mp. |
| 12 | ((deltoid or pectoral or chest wall or thoracic or thoraco-lumbar or thoracolumbar or lumbosacral or lumbo-sacral or lumbo-pelvic or lumbopelvic or lumbo-ischial or lumbar-ischial or sterno-clavicular or sternoclavicular or sternocleidomastoid or scapulohumeral or scapulo-humeral or intercostal or inter-costal or claviculosternal or claviculo-sternal or sternocostal or sterno-costal or costosternal or costo-sternal or costoclavicular or costo-clavicular or dorso-lumbal or lumbal or zygapophyseal or arm*1 or upper limb* or upper extremit* or shoulder* or acromial or scapula* or brachium or brachial or antebrachium or antebrachial or elbow* or cubital or antecubitis or antecubital or olecranon or olecranal or forearm* or wrist* or carpus or carpal or hand*1 or manus or palm or palma or palmar or finger* or digit* or phalanges or phalangeal or thumb* or pollex or subacromial or sub-acromial or acromioclavicular* or acromio- clavicular* or metacarpocarpal or metacarpo-carpal or carpometacarpal or carpo-metacarpal or metacarpophalangeal or metacarpo-phalangeal or interphalangeal or inter-phalangeal or radioulnar or radio-ulnar or radio-carpal or radiocarpal or radiohumeral or radio-humeral or glenohumeral or humeroscapular or gleno-humeral or humero-scapular or scapulo-humeral or scapulohumeral or intercarpal or inter-carpal or midcarpal or mid-carpal or intermetacarpal or inter-metacarpal or humeroradial or humero-radial or humeroulnar or humero-ulnar or ulnohumeral or ulnocarpal) adj (pain* or ache*)).mp. |
| 13 | ((hip*1 or hipbone* or lower limb* or lower extremit* or leg*1 or thigh* or femur or femoral or knee* or patella* or popliteus or popliteal or tibial or shin* or crus or crural or calf or sura or sural or fibular or ankle or tarsus or tarsal or heel or calcaneus or calcaneal or feet or foot or forefoot or midfoot or hindfoot or pes or pedal or planta or plantar or digit* or phalanges or phalangeal or toe*1 or hallux or acetabulofemoral or acetabulo-femoral or tibiofemoral or tibio-femoral or patellofemoral or patello-femoral or astragalocrural or astragalo-crural or talo-crural or talocrural or metatarsophalangeal or metatarso-phalangeal or intertarsal or inter-tarsal or tibiotarsal or tibio-tarsal or tibiotalar or tibio-talar or talocalcanea* or talo-calcanea* or subtalar or sub-talar or tibiofibular or tibio-fibular or talonavicular or talo-navicular or tarsometatarsal or tarso-metatarsal or achilles or trochanteric) adj (pain* or ache*)).mp. |
| 14 | ((cartilage* or fascia* or myofascial or trigger point or ligament* or tendon* or joint* or locomotor or tissue) adj (pain* or ache*)).mp. |
| 15 | ((intervertebral or disc or disk or paraspinal or hamstring or pectoralis or psoas or quadriceps or rotator cuff or iliopsoas) adj (pain* or ache*)).mp. |
| 16 | ((skeletal or bone* or vertebra* or metatarsal or intermetatarsal or metacarp* or talus or fibula or tibia or humerus or radius or ulna* or phalanx or clavicle or sesamoid or trochanter* or iliac or ischiogluteal or ischial) adj (pain* or ache*)).mp. |
| 17 | or/1-16 |
| 18 | CIRCUIT-BASED EXERCISE/ or ENDURANCE TRAINING/ or HIGH-INTENSITY INTERVAL TRAINING/ or RUNNING/ or JOGGING/ or SWIMMING/ or WALKING/ or STAIR CLIMBING/ or DANCING/ or BICYCLING/ or BOXING/ or DANCE THERAPY/ or WATER SPORTS/ or PHYSICAL ENDURANCE/ or PHYSICAL EXERTION/ or PHYSICAL FITNESS/ or CARDIORESPIRATORY FITNESS/ or ATHLETIC PERFORMANCE/ or "Physical Education and Training"/ or EXERCISE TEST/ or WALK TEST/ or EXERCISE THERAPY/ or EXERCISE TOLERANCE/ or ANAEROBIC THRESHOLD/ |
| 19 | (aerobic dance or aerobics or aerobic sport* or aerobic training or aerobic exercise* or aerobic interval training or intensity aerobic activit* or systematic exercise* or endurance train* or endurance exercis* or intensity train* or intensity exercis* or acute exercis* or circuit train* or kinetic chain exercis* or active exercis* or dynamic exercis* or open chain exercis* or high-intensity intermittent exercis* or high-intensity intermittent training or high-intensity interval exercis* or high-intensity interval training or sprint interval train* or climbing stairs or stairclimbing or stair climb* or treadmill exercise or treadmill training or bicycling or aerobic cycling or walking or jogging or running or swimming or dancing or weight-bearing exercis* or hiking or athletics or endurance sport* or aquatic sport* or triathlon* or rowing or aquatic exercis*).mp. |
| 20 | (physical endurance or physical fitness or cardiorespiratory fitness or cardio-respiratory fitness or physical training or physical exertion* or ergomet* test* or ergomet* exercis* or cardiopulmonary exercis* or exercise test* or fitness test* or step test* or treadmill test* or endurance test* or walk test* or movement therap* or dance therap* or exercise therap* or rehabilitation exercis* or remedial exercis* or physical activit* regimen* or physical activit* program* or physical activit* train* or exercise tolerance or an?erobic threshold*).mp. |
| 21 | (therapeutic exercise* or exercise therap* or exercise treatment or prescribed exercise* or dance therap* or exercise test* or walk* test*).mp. |
| 22 | (cardio* exercis* or cardio* training or cardio machine* or spinning or cross country skiing or boxing or kickboxing or biking or cardio* workout* or aerobic workout* or exercise workout* or endurance workout* or acute workout* or physical workout* or fitness workout* or aerobic gym exercis* or aerobic gym workout* or zumba or water workout* or cycle class* or cycle exercis* or exercise bicycle or exercise bike or spinning bike or exercycle or elliptical trainer or cross-trainer or skipping rope or rope skipping or jumping rope or rope jumping).mp. |
| 23 | or/18-22 |
| 24 | 17 and 23 |
| 25 | Central Nervous System Sensitisation/ |
| 26 | pain perception/ or nociception/ |
| 27 | (Sensiti#ation or sensitivity or hypersensitivity or hyperexcitability or hyperalgesi* or hyperalgi* or hyperpathi* or hyper?esthesi* or hyposensitiv* or hypalgesi* or algesi* or algesthesis* or neural inhibition or nociception* or nocipercepti* or nociceptive* or algometry or allodynia).mp. |
| 28 | (pain adj3 (modulat* or threshold* or process* or pathophysiolog* or perception or perceiv* or inhibit*)).mp. |
| 29 | (pain adj2 (tolerance* or tolerat* or sense or sensing or sensation*)).mp. |
| 30 | 25 or 26 or 27 or 28 or 29 |
| 31 | 24 and 30 |
| 32 | (exercise adj4 (hypoalgesi* or analgesi* or hypo-algesi*)).mp. |
| 33 | (pain* adj4 (reduc* or relief or reliev* or diminish* or intens*) adj7 exercis*).mp. |
| 34 | 17 and 33 |
| 35 | 31 or 32 or 34 |
| 36 | limit 35 to english language |
| 37 | limit 36 to (case reports or editorial or letter or news) |
| 38 | 36 not 37 |
| 39 | (pregnan* or gestation* or antenatal or prenatal or natal or gravidit* or gravida* or multigravid* or primigravid*).mp. |
| 40 | 38 not 39 |
| 41 | (newborn* or neonat* or infant* or infancy or preschool* or pre-school*).mp. |
| 42 | 40 not 41 |
| 43 | limit 42 to ("newborn infant (birth to 1 month)" or "infant (1 to 23 months)" or "preschool child (2 to 5 years)" or "child (6 to 12 years)") |
| 44 | 42 not 43 |
